# Conference Report: Review of Clinical Implementation of Advanced Quantitative Imaging Techniques for Personalized Radiotherapy

**DOI:** 10.3390/tomography10110132

**Published:** 2024-11-14

**Authors:** Yevgeniy Vinogradskiy, Houda Bahig, Nicholas W. Bucknell, Jeffrey Buchsbaum, Hui-Kuo George Shu

**Affiliations:** 1 Department of Radiation Oncology, Thomas Jefferson University, Philadelphia, PA 19107, USA; 2 Department of Radiology, Radiation Oncology and Nuclear Medicine, Centre Hospitalier de l’Universite de Montreal (CHUM), Montreal, QC H2X 3E4, Canada; 3 Sir Charles Gairdner Hospital, Nedlands 6009, Australia; 4 National Cancer Institute, Bethesda, MD 20892, USA; 5 Department of Radiation Oncology, Winship Cancer Institute of Emory University, Atlanta, GA 19104, USA

**Keywords:** quantitative imaging, dual energy, image-guided radiation therapy response-based, magnetic spectroscopy

## Abstract

The topic of quantitative imaging in radiation therapy was presented as a “Masterclass” at the 2023 annual meeting of the American Society of Radiation Oncology (ASTRO). Dual-energy computed tomography (CT) and single-positron computed tomography were reviewed in detail as the first portion of the meeting session, with data showing utility in many aspects of radiation oncology including treatment planning and dose response. Positron emission tomography/CT scans evaluating the functional volume of lung tissue so as to provide optimal avoidance of healthy lungs were presented second. Advanced brain imaging was then discussed in the context of different forms of magnetic resonance scanning methods as the third area noted with significant discussion of ongoing research programs. Quantitative image analysis was presented to provide clinical utility for the analysis of patients with head and neck cancer. Finally, quality assurance was reviewed for different forms of quantitative imaging given the critical nature of imaging when numerical valuation, not just relative contrast, plays a crucial role in clinical process and decision-making. Conclusions and thoughts are shared in the conclusion, noting strong data supporting the use of quantitative imaging in radiation therapy going forward and that more studies are needed to move the field forward.

## 1. Introduction

Radiation therapy has historically been a cancer treatment modality that relies on computed tomography (CT) [1] and magnetic resonance imaging (MRI) [2] anatomical-based imaging to generate radiation treatment plans. In the last 20 years, positron emission tomography (PET) imaging using fluorodeoxyglucose has further improved the ability of radiation oncologists to help target tumors [3]. Recently, there have been imaging-based developments that have enabled the integration of quantitative, functional-based imaging into the radiotherapy (RT) clinical paradigm [4,5,6,7].

The functional imaging methods integrated into RT span a wide range of imaging modalities including MRI [7], CT [4,6], and PET/CT imaging [5], providing information on both the tumor and normal tissues, and have been incorporated into the RT treatment of a variety of disease sites including the lung [6], brain [8], head and neck, and liver [9]. The hypothesis is that the incorporation of functional imaging in the RT clinical paradigm can provide spatial, functional information on either the tumor response or organ (normal tissue) function and that these data can then be used to improve clinical outcomes (either an improvement in tumor control or a reduction in toxicity).

The functional imaging in RT research has progressed from proof of concept of the functional imaging modalities [10,11] to validation of the functional imaging modalities against existing methods and established clinical metrics [12,13] to the demonstration of the clinical utility of functional imaging [8,14]. In the last 5 years, numerous prospective, early-phase clinical trials have been conducted directly incorporating functional imaging to guide RT. Examples of ongoing or recently concluded clinical trials include NCT02528942, NCT05302817, NCT05134558, NCT02773238, NCT02492867, NCT0313788, NCT01445691, NCT04863027, and NCT03569072. Early data from these and other clinical trials suggest promising clinical outcomes may be achievable by incorporating functional imaging into the RT paradigm [4,15,16].

Incorporating functional imaging into RT clinical practice has highlighted unique challenges, including managing the uncertainties related to the functional imaging modality, finding robust workflows to incorporate functional imaging in busy clinical RT environments, developing quality assurance (QA) methods, and finding pertinent and feasible clinical end-points that can robustly demonstrate the clinical utility of functional imaging. To present the exciting clinical utility and development of functional imaging in RT and highlight the challenges associated with functional imaging in RT, the American Society for Radiation Oncology (ASTRO) and National Institute of Health group on Quantitative Imaging in Radiotherapy (QIRT) organized a Master Class session at the 2023 national ASTRO meeting titled, ‘Master Class: Advanced Quantitative Imaging Techniques for Personalized Radiotherapy and Lung Toxicity Avoidance’. The ASTRO Master Class occurred on 31 September 2023 in San Diego, California. The talks for the Master Class included: (1) Dual-Energy CT and SPECT for Functional Lung Assessment, (2) Implementing of Quantitative Imaging Modalities for Brain Tumors to Guide RT Targeting, (3) V/Q PET for Functional Lung Assessment and Avoidance, (4) Development of Personalized Radiotherapy Techniques through the Incorporation of Novel Imaging Methodologies, and (5) QA Issues with Widespread Implementation of Quantitative Imaging Modalities. The purpose of this work is to summarize the ASTRO and QIRT conference session on quantitative imaging in RT. Topics covered will include dual-energy CT (presented by co-author H.B.) and PET-CT for functional lung avoidance (presenting by co-author N.W.B.), functional MRI imaging for improved RT treatment of brain tumors (presented by co-author H.G.S.), development of personalized radiotherapy through the incorporation of novel imaging (presented by Dr. Clifton Fuller and section written by co-author H.G.S.), and a summary of QA methods needed for functional imaging-based RT (presented by Dr. Michael Knopp, written by co-author Y.V.).

## 2. Conference Sections

### 2.1. Dual-Energy CT and SPECT for Functional Lung Assessment

Radiation therapy (RT) pulmonary toxicity encompasses a spectrum from acute injury, characterized by damage to alveolar epithelium and increased endothelial permeability to chronic inflammation culminating in permanent fibrosis [17]. The fibrosis process involves the release of pro-inflammatory cytokines, active immune cells, chemokines, and growth factors [18,19]. Symptomatic radiation pneumonitis is observed in 10–30% of cases with locally advanced lung cancer and 2–5% in early-stage lung cancer, and pneumonitis can prove fatal in some rare instances [20,21]. Predicting radiation pneumonitis (RP) is complex. Previous studies indicate that both patient-related factors (age over 60, smoking habits, lower lung lobe location, poor baseline function, and interstitial lung disease) and treatment-related factors (high dose radiation therapy, large radiation volume, hypofractionation, concurrent chemotherapy) significantly influence RP and fibrosis development [22,23,24,25]. Dosimetric parameters such as mean lung dose (MLD) and percent lung volume receiving specific doses show variable predictive power for RP, with quantitative analysis of normal tissue effects in the clinic (QUANTEC) indicating a linear increase in RP risk with dose [24].

Given that up to 70% of lung cancer patients have concurrent lung disease [26], and lung function is not uniform, RT planning that considers individual lung function variability has the potential to reduce toxicity. Potential intervention points include precise lung dose assessment and personalized functional avoidance during planning, functional RT adaptation during treatment, and post-treatment strategies such as toxicity prediction and early intervention for immunotherapy-induced pneumonitis. Lung function sparing RT planning is particularly relevant for patients with borderline pulmonary function, large RT fields, re-irradiation, or dose-escalation contexts. The standard lung function assessment method is perfusion single-photon emission computed tomography (SPECT), which uses Technetium-99m-labeled macroaggregated albumin [27,28]. It is deemed more sensitive than ventilation assessments, as decreased ventilation generally leads to reduced perfusion, but the inverse is not necessarily true [29]. However, SPECT has limitations, including poor spatial and temporal resolution and inconsistency in patient setup and breathing regimes. Emerging methods for functional sparing RT, such as hyperpolarized MRI [30,31], perfusion MRI [32], ventilation 4-dimensional computed tomography (4D-CT) [4,33,34], gallium-68 ventilation/perfusion (V/Q) position emission tomography-CT (PET-CT) [5,13], xenon-enhanced CT [35,36], and perfusion duel energy (DECT) [10], are under investigation.

**Figure 1:**
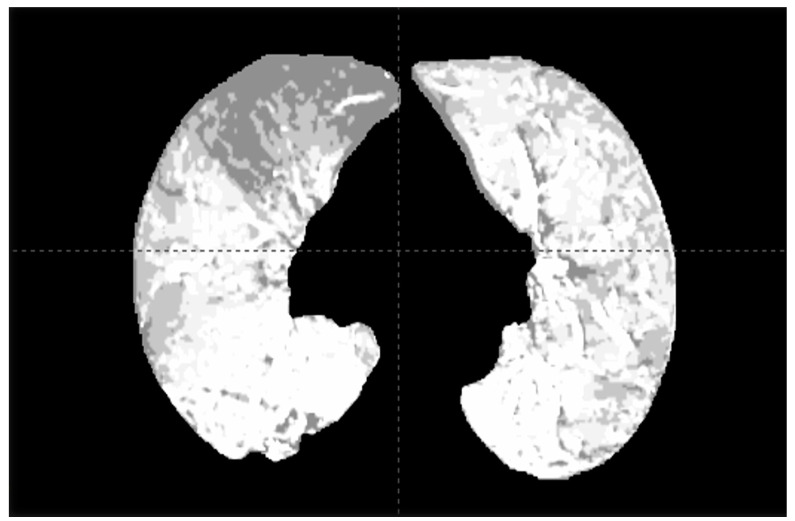
Iodine partial electronic density to derive a regional blood volume map.

DECT leverages X-rays at two distinct energies to facilitate material decomposition [37]. This advanced technique enables detailed tissue characterization based on differential attenuation, utilizing the interplay between the photoelectric effect and the energy level of each element. The dual-energy process is crucial for quantifying substances like iodine or calcium. The significance of DECT in RT, especially for perfusion, stems from its capacity to offer comprehensive tissue characterization information without requiring additional exams [6]. DECT eliminates the need for image fusion and provides a superior assessment of lung morphology and texture. Moreover, it is dose-neutral when compared to standard planning CT, making it an efficient tool in RT planning [6,38]. In a first prospective study assessing the role of DECT-derived iodine maps (Figure 1) in patients undergoing radiation therapy for lung cancer, and the dosimetric implications of integrating DECT into the RT planning process, thoracic DECT scans in the treatment position were utilized to determine the iodine fraction in lung voxels, representing regional blood volume, to derive segmented lung function [6]. Twenty-five patients receiving stereotactic body radiation therapy (SBRT) for early-stage or intensity-modulated radiation therapy (IMRT) for locally advanced lung cancer, with a majority having chronic obstructive pulmonary disease, were enrolled (NCT04863027). The study focused on analyzing each voxel’s contribution to total lung function, based on iodine distribution, through a two-material decomposition approach [10]. The DECT-derived lobar function was then compared with SPECT/CT. Incorporating a functional map into the treatment planning, using six subvolumes with varying levels of iodine distribution, allows for a comparison between the percentage of lung volume receiving 5 Gy (V5), V20, and MLD to anatomical versus functional lungs [6]. The correlation between DECT- and SPECT/CT-derived lobar function was strong, indicating significant differences in V5 and MLD between anatomic and functional lung volumes. This correlation underscores the potential of DECT in enhancing lung function-sparing in radiotherapy. In practice, functional information was found to be most crucial for patients with anatomically heterogeneous lung function. In these cases, traditional dose-volume metrics might either underrepresent the lung dose, leading to an underestimated toxicity risk or overrepresent it, potentially causing unnecessary clinical compromises or adverse decisions. A further aspect of this study focused on using DECT to evaluate longitudinal lung function changes over time in lung cancer patients treated with radiotherapy [39]. Forty-eight patients were prospectively enrolled, undergoing contrast-enhanced DECT scans before treatment and at 6 and 12 months post-treatment (NCT04863027) [40]. The study aimed to correlate changes in normalized functional responses (NFR), calculated for different dose ranges, with pulmonary function test (PFT) results. The findings revealed a linear correlation between NFR and dose, with the time elapsed post-radiotherapy also correlating with NFR, though this was not observed in the stereotactic body radiation therapy subgroup. Interestingly, no correlation was found between NFR and changes in PFT, highlighting the potential of DECT-derived iodine maps for detailed anatomical evaluation of radiation’s impact on lung function, including subtle changes.

The integration of functional information in RT, specifically in IMRT for locally advanced disease and SBRT for early-stage disease, is still in the process of being validated. A review of six clinical studies highlighted the benefits of using perfusion SPECT for lung RT planning [41]. It was found that functional dosimetry is especially helpful for patients with emphysema or notable perfusion deficits, and it helps in more accurately estimating the risk of RP. These advantages are particularly evident in patients with borderline pulmonary function, extensive radiation therapy fields, re-irradiation cases, or during dose-escalation treatments. A recent Phase 2 multicenter clinical trial evaluated 4DCT-ventilation functional avoidance RT [4]. The study hypothesis was that functional avoidance RT could reduce the rate of grade ≥2 radiation pneumonitis to 12% compared with a 25% historical rate, with the trial being positive if ≤16.4% of patients experienced grade ≥2 pneumonitis. Participants included 67 lung cancer patients receiving curative-intent RT and chemotherapy treated to a dose of 60 Gy, delivered over 30 fractions. The study used 4DCT scans to create ventilation images, which then guided the formulation of functional avoidance plans, minimizing doses to functional lung areas while maintaining prescribed tumor doses. The functional avoidance approach achieved an average reduction of 3.5% in the functional lung volume receiving ≥20 Gy. The rate of grade ≥2 radiation pneumonitis was 14.9%, which met the criteria for the phase 2 study, suggesting that 4DCT ventilation warrants further evaluation in a phase 3 study.

Lung function avoidance methods in RT planning are not without limitations. They may provide functional information with large uncertainties in certain clinical scenarios, necessitating cautious interpretation and a thorough review of anatomical images. For instance, previously irradiated lung areas, considered as avoidance structures, may pose a risk of abscess and necrosis. This necessitates a careful balance against the risk of RP. Other organs at risk, like the heart, bronchus, or esophagus, must also be considered. Clinical scenarios that complicate decision-making include post-obstructive pneumonia caused by central tumors affecting perfusion and ventilation, and perfusion defects in interstitial lung disease, where it is challenging to determine whether inflammation or fibrotic areas are more problematic. Similarly, assessing longitudinal changes in lung function after radiotherapy using DECT or other methods is feasible but presents several challenges. These include the need for image registration due to varying patient positions and the complexities of image transformation processes, such as rigid transformation and diffeomorphism. Regarding iodine concentration in DECT, normalization against a reference volume is necessary for an accurate assessment of functional response. Standardization of DECT iodine maps and other lung function assessment methods is further complicated by inter- and intra-patient variability. Parameters such as the amount of contrast, acquisition time, and iodine map values need uniform guidelines. While iodine maps predominantly measure perfusion, rare cases of normal perfusion with low ventilation present diagnostic challenges. Finally, organizational hurdles, including managing an increased number of sequences for data storage and the learning curve for medical teams, are significant considerations.

### 2.2. V/Q PET for Functional Lung Assessment and Avoidance

An alternative lung function imaging technique that has been proposed for lung function assessment and avoidance is 4D V/Q PET/CT using a Galium-68 tracer (^68^Ga-4D-V/Q PET/CT). The ^68^Ga-4D-V/Q PET/CT technique is a promising functional lung imaging technique that has a number of advantages over other types of functional lung imaging including high spatial accuracy and capacity to account for respiratory motion [42]. Disadvantages of ^68^Ga-4D-V/Q PET/CT include that the technique involves an additional PET scan and that the radiotracers are investigational and do not have regulatory approval for use in some countries. ^68^Ga-4D-V/Q PET/CT has been compared with dual-energy CT perfusion with a strong correlation between the modalities observed (r_p_ = 0.98 and *p* < 0.001). CT ventilation and ^68^Ga-4D-V/Q PET/CT have also been compared and less correlation was observed between the two modalities (r_s_ 0.52 ± 0.10) [12,43].

The ^68^Ga-4D-V/Q PET/CT technique is performed in a single 40-min procedure as a radiation therapy planning scan. Ventilation is imaged with the patient inhaling 5 MBq of ^68^Ga-Galligas (Ga68 nanoparticles) followed by a 4D-CT, and subsequently, a respiratory-gated ventilation PET is acquired [42]. Without the patient moving, 20–40 MBq^68^Ga-MAA is injected intravenously followed by the perfusion respiratory gated PET acquisition [42]. The 4D-CT acquired at the time of ^68^Ga-4D-V/Q PET/CT is used for both attenuation correction and radiation treatment planning.

Correlation with ^68^Ga-4D-V/Q PET/CT and pulmonary function tests suggests that the V/Q matched region between 15% and 35% of function provides the highest correlation with pulmonary function measures [44]. In ^68^Ga-4D-V/Q PET/CT studies, the functional lung volumes are created by first excluding artifacts, then selecting all lungs with radiotracer uptake equal to or greater than 30% of the maximum threshold for ventilated and perfused lungs, respectively, and then finding the overlap of these regions.

Performing ^68^Ga-4D-V/Q PET/CT imaging before and after treatment has enabled a dose-response relationship to be established that approximates a loss of perfusion of 0.75% per Gy and a loss of ventilation of 0.71% per Gy [45]. Changes in functional volumes between pre-treatment and mid-treatment imaging have been assessed. Repeat imaging in the 4th week of a 6-week treatment has demonstrated that a majority of patients experience a mean decrease in their perfused lung volume and a mean increase in their ventilated lung volume [5]. A study of 25 patients found that the perfused lung volume decreased in 16 patients with a mean change in volume (cc) of −28 ± 515 cc and the ventilated lung volume increased in 13/25 patients with a mean of the change in volume (cc) +112 ± 590 cc [5].

The high-intensity functional image-guided VMAT lung evasion (HI-FIVE) trial (NCT03569072) was conducted in patients with stage III NSCLC undergoing chemoradiotherapy to assess the feasibility of using ^68^Ga-4D-V/Q PET/CT to adapt radiation treatment plans to avoid functional lung. Eighteen patients were recruited and 15 met the criteria for feasibility [46]. The average reduction in functional MLD was 12.4% (SD ± 12.8%) and the average reduction of function V20 was 22.9% (SD ± 11.9%) [46]. Toxicity and progression-free survival were acceptable and comparable to recent studies such as the PACIFIC trial.

Despite the promising early-phase trial data, the exact clinical benefits of performing functional lung adaptation are unknown. Toxicity probability modeling suggests that some individual patients could benefit from as much as a 50% reduction in the rate of high-grade lung toxicity with average reductions in grade 2 + RP of 5–6% and grade 3 + RP by between 2 and 4% [14]. However, more work is needed to establish if these projected benefits translate into clinical benefits for patients. The ideal functional modality is yet to be established and studies comparing different imaging modalities will enable future trials to be designed using the most effective and practical modality.

### 2.3. Implementation of Quantitative Imaging Modalities for Brain Tumors to Guide RT Targeting

Conventional MRI, particularly the T1w-contrast enhanced (CE) sequence, can generally determine the full extent of brain metastases and meningiomas but is less capable for glioblastomas (GBMs), which are infiltrative beyond enhancing tumors. While T2 with FLAIR sequences have been used to identify GBM infiltration, the sequence is neither sensitive enough to find all infiltration nor specific enough to distinguish infiltration from edema. Thus, conventional MRI may be insufficient for RT planning of GBMs. More advanced quantitative imaging modalities may improve GBM target delineation. Here, we present proton magnetic resonance spectroscopic imaging (MRSI) as a use case for what is needed to develop and validate this modality to improve RT targeting.

MRSI performed on standard MRIs obtains spectra of evaluated volumes that can determine levels of higher abundance metabolites. Changes in these metabolites have been associated with GBM tumors including increased choline (Cho) and decreased N-acetylaspartate (NAA) levels [47,48]. With specific metabolite signatures for GBMs and normal brains, MRSI may help define infiltrating tumor extent not appreciated on conventional MRI for RT planning [49,50,51,52]. Current implementations of MRSI that evaluate single voxels or multiple voxels on a single plane are limited with low resolution (e.g., 1–2 cc) and poor signal-to-noise ratio, particularly if performed on 1.5T scanners. A more advanced form of MRSI (requiring modern 3T scanners) that can assess the entire brain at high resolutions (down to 0.1 cc) within a reasonable time (approximately 14 min) has been developed which we have explored to aid the targeting of GBM with RT [7,53]. We have termed this more advanced scan as spectroscopic MRI (sMRI) to distinguish it from previous implementations of MRSI [7,54].

**Figure 2:**
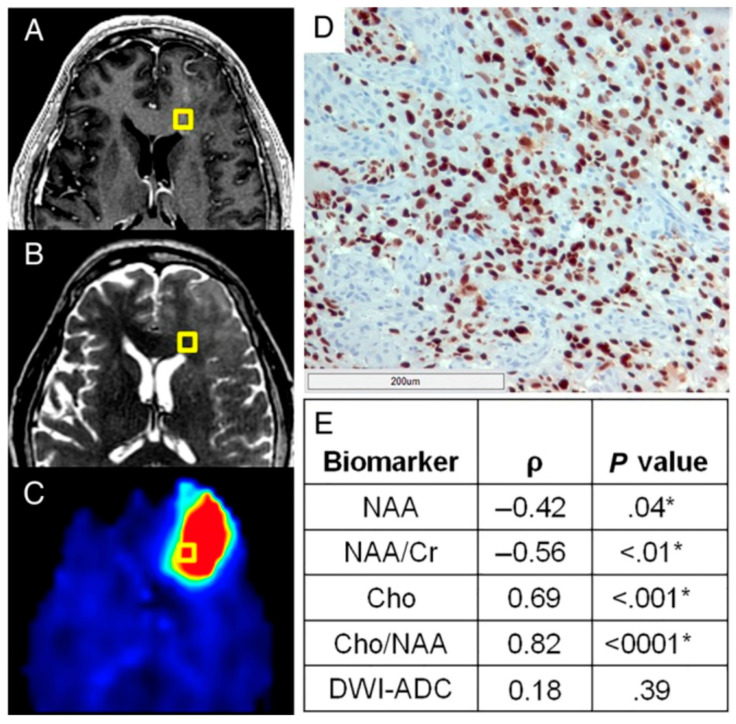
A normalized metric of tumor infiltration, Sox2 density, identifies tumors outside of conventional imaging and exhibits striking correlations with sMRI biomarkers. Though no obvious abnormality can be found on preoperative T1w-CE (**A**) or T2w imaging (**B**) in this patient, a striking elevation in Cho/NAA (**C**) on sMRI suggests substantial tumor infiltration. (**D**) A light micrograph of tissue (including a 200 μm scale bar) from the gold box showed elevations in Sox2 density along with the microvascular proliferation and nuclear atypia suggestive of GBM. (**E**) Statistically significant correlations were seen between various normalized metabolic markers and Sox2 density, with Cho/NAA exhibiting the strongest association. * Significant at *p* < 0.05. DWI-ADC, diffusion-weighted image–apparent diffusion coefficient. Originally published in the following reference [7].

To establish sMRI as a new modality for guiding RT targeting of GBM, we correlated image findings with pathology as well as patterns of recurrence. Toward these goals, we conducted a study of GBM patients who underwent preoperative conventional MRI and sMRI (NCT01445691) [7]. Stereotactic biopsies were obtained around regions of enhancing tumors prior to full tumor resection with biopsy locations mapped on both conventional MRI sequences as well as sMRI metabolite maps. Of the assessed sMRI metabolites, the Cho/NAA ratio was most strongly correlated with the density of GBM tumor cells within biopsy specimens (Figure 2). Next, pre-RT sMRI scans were obtained on a cohort of 11 GBM patients receiving standard RT guided by conventional MRI [8]. First recurrence sites were identified and compared with true RT target volumes as well as target volumes modified by elevated Cho/NAA on sMRI. Results show that significant regions of infiltrative tumor identified by sMRI were outside of standard high-dose targets determined and that coverage of regions of 1st recurrence would have been significantly improved if sMRI had been used to aid target definition. Of note, Cho/NAA in tumors were normalized to average Cho/NAA of the contralateral normal-appearing white matter (NAWM) to improve consistency across patients. Based on these studies, we found that Cho/NAA values 2 times contralateral NAWM were a good threshold for defining regions of infiltrative GBM with high recurrence risk [7,8,16].

**Figure 3:**
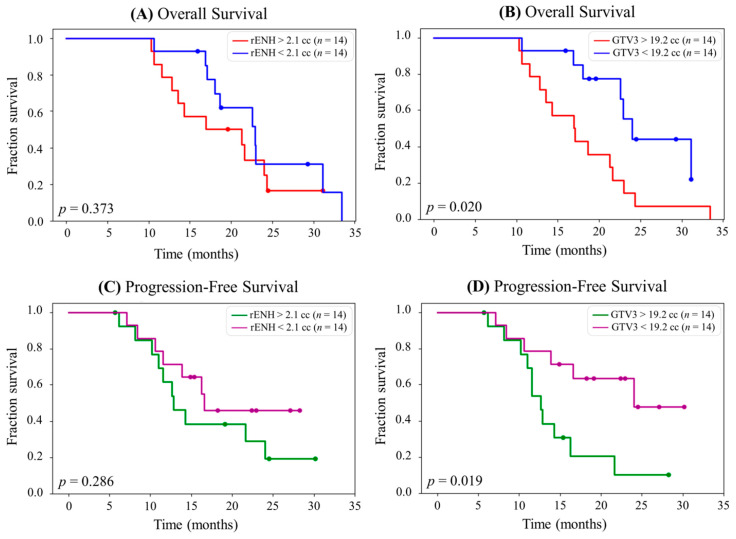
(**A**) Kaplan–Meier survival curve for overall survival (OS) stratified into equal groups by the median residual contrast-enhancing (rENH) tumor volume (2.1 cc). The median OS for patients with rENH > 2.1 cc is 21.3 months, and the median for OS for patients with rENH < 2.1 cc is 23.0 months. Of note, the assessment only included 28 of 30 patients from the study (2 were excluded due to harboring IDH1 mutation) (**B**) Kaplan–Meier survival curve for OS stratified into equal groups by the median gross tumor volume 3 (GTV3) (19.2 cc). The median OS for patients with GTV3 > 19.2 cc is 17.1 months, and the median OS for patients with GTV3 < 19.2 cc is 27.4 months. (**C**) Kaplan–Meier survival curve for progression-free survival (PFS) stratified into equal groups by the median rENH tumor volume (2.1 cc). The median PFS for patients with rENH > 2.1 cc is 12.8 months, and the median PFS for patients with rENH < 2.1 cc is 19.0 months. (**D**) Kaplan–Meier survival curve for PFS stratified into equal groups by the median GTV3 (19.2 cc). The median PFS for patients with GTV3 > 19.2 cc is 12.6 months, and the median PFS for patients with GTV3 < 19.2 cc is 24.0 months. Originally published in the following reference [60].

To clinically test sMRI for aiding GBM target delineation, a 3-site clinical trial of sMRI-guided RT dose escalation for newly diagnosed GBMs was conducted (NCT0313788). Prior to this study, custom software was developed (Brain Imaging Collaboration Suite, BrICS) to enhance the usability of sMRI allowing clinicians to utilize sMRI data in a more user-friendly environment and implement its use for this study [16,55]. The trial study targeted regions of Cho/NAA > 2 times NAWM plus residual enhancing tumors with 75 Gy in 30 fractions while typical tumor volumes contoured based on T1w-CE and T2w/FLAIR sequences were still targeted to 60 and 50 Gy in 30 fractions, respectively. With 30 patients, sMRI-guided dose-escalated RT was found to be both feasible and tolerable [16]. Median progression-free survival (PFS) and overall survival (OS) were 16.6 and 23.3 months [16], respectively, which compares favorably with standard treatment in recent phase III studies as well as with an RT dose-escalation study conducted by NRG Oncology (NRG-BN001) that used only standard MRI for tumor definition with median OS in the 16.0–18.7 months range [56,57,58,59]. Supporting our assertion that elevated Cho/NAA is superior to residual enhancement as an imaging biomarker, we found that higher residual 2x Cho/NAA volumes were more predictive of worse survival than higher residual enhancing tumor volumes (Figure 3) [60].

A French randomized study (SPECTRO GLIO trial) was recently reported that used MRSI (albeit a less advanced form than sMRI) to guide RT dose escalation for GBM to 72 Gy in 30 fractions (the trial evaluated 180 patients) and did not find improved survival for the high dose arm [61]. While the trial was an impressive effort to harmonize the use of an advanced quantitative imaging modality across several institutions, an editorial was published on the trial challenges that contributed to the negative result [62].

Other advanced imaging approaches are also currently proceeding along a similar pathway as sMRI for validation/implementation. ^18^F-DOPA PET and diffusion/perfusion imaging are additional modalities capable of identifying infiltrative GBM not seen on conventional MRIs [63,64]. Similar to sMRI, single-site trials using these imaging modalities to guide RT dose escalation for newly diagnosed GBMs have produced similarly promising results but cross-institutional validation of translatability for these efforts is still needed [65,66].

In summary, we have reviewed our use case of how to approach, validate, and implement sMRI for GBM imaging. This example can serve as a roadmap to establish new advanced quantitative imaging modalities for clinical use. Based on the myriad of more advanced imaging modalities being explored for GBM, there are likely to be advances in how to best define RT targets for this diagnosis in the very near future.

### 2.4. Development of “Personalized Radiotherapy” Techniques Through the Incorporation of Novel Imaging Methodologies

The simultaneous imaging of not only anatomy but of functional and spatial alteration and motion of both tumor and normal tissue over time is needed to advance image guidance for RT. To be truly useful, the imaging data needs to fulfill the following 4 A’s: (1) anticipatory (predictive/early), (2) actionable (potential to change care), (3) accurate (in time and 3D space) and (4) additive (consider more than one feature or function at a time). How radiation treatments are approached has already changed based on increased use of imaging and the trend of increased use of imaging in RT is continuing. Previously, radiation oncology transitioned from using static anatomic fields to static anatomic volumes (IMRT) in the late 1990sؘ–early 2000s with better imaging of both tumor and normal tissues. Currently, we have changed from using static anatomic volumes to moving anatomic volumes, known as image-guided radiotherapy (IGRT), which accounts for moving tumors and normal structures. Still ongoing is the next move of using functional or biologic volumes (B-IGRT) where advanced imaging can better define tumor and normal tissues before, during, and after treatment. These transitions will be illustrated with head and neck (HN) cancers as the use case.

An early adaptive RT (ART) approach was implemented where serial CT scans were obtained on patients with HN cancer during treatment. This approach identified significant shifts to normal structures due primarily to weight loss in patients and adaptive replanning to account for these shifts resulted in an apparent reduction of toxicities [67]. However, this type of result was not necessarily reproducible with a more recent French multi-institutional study randomizing patients with HN cancer to adaptive RT (with weekly replanning CTs to spare parotids) versus standard IMRT finding no difference in xerostomia defined by stimulated salivary flow ≤ 500 mg/min [68]. These inconsistent results may have been influenced by the aggressiveness of adaptation to detected shifts in normal structures. In addition, these implementations of ART illustrate the limitations of imaging techniques such as non-contrast CT which generally does not permit adaptation to tumor due to inadequate visualization.

Alternative imaging modalities such as MRI can greatly improve tumor visualization. Mohamed et al. reported the experience tracking HPV+ oropharyngeal cancer patients serially with MRIs during RT and found that half had a complete response (CR) and one-third had > 50% partial response (PR) by midway through RT [69]. These findings raise interesting questions regarding how to perform tumor adaption in situations with significant tumor response including a CR with an approach that is currently being tested on our institutional MR-ADAPTOR phase II trial (NCT03224000).

Moving on to utilizing advanced imaging to provide functional/biologic information on tumors during RT, the group has started to perform diffusion-weighted MRI (DWI) for this purpose in patients with HN cancer. DWI has some significant advantages including simplicity of acquisition, lack of need for contrast, being quantitative, and finally, being standardizable against a reference phantom. Multiple previous reports have shown that early DWI changes during RT can be predictive of outcome [70,71]. However, significant technical challenges remain in using DWI for RT targeting because, quite often, sequences that provide the best signal-to-noise ratio (SNR) and the highest sensitivity and specificity for tumor detection may have significant spatial distortions. Such DWI sequences may still be favored by diagnostic radiologists but would be inadequate for radiation oncologists who require the highest degree of geometric accuracy. Schakel et al. found in testing various DWI sequences that an alternative turbo spin echo sequence termed DW-SPLICE showed virtually no image distortion while still providing an adequate SNR for tumor assessment [72]. We have now tested DW-SPLICE compared with alternatives and validated its performance in a series of HN cancer patients [73]. One important consideration is that being able to consistently acquire quantitative images serially may be more important than having the highest SNR for individual scans which is illustrated by our work showing the ability to detect significant trends for change with tumor apparent diffusion coefficient (ADC) values over an RT course whereas changes between scans from individual time points showed only negligible differences [74]. Additional important concepts to consider are illustrated by the work evaluating DWI as a potential biomarker of outcomes in patients with HN cancer where tumor volume changes over time raises questions regarding how subvolumes need to be evaluated if they change substantially during treatment and how to choose rational discriminant biomarker values within tumor volumes that can serve as a predictor of outcome [75].

Given the rapid progress in the development of advanced quantitative imaging, we will no doubt see an acceleration in its use as a potential biomarker for image-guided ART. Highlighting some of the state-of-the-art research in this area by others in the HN cancer field, advanced imaging can help better identify tumor regions to permit dose escalation of higher-risk regions or dose de-escalation of lower-risk regions or a combination of the two. The first example is a study from China that randomized 260 patients with stage III-IVa nasopharyngeal carcinoma where the control group had GTVs treated to standard RT doses of 70.4–72.6 Gy in 32–33 fractions while the experimental group had a boost of a subvolume of the GTV with ADC values from DWI below the mean of the entire GTV to doses of 75.2–77.55 Gy in 32–33 fractions. With this dose intensification, the experimental group had a statistically significant improvement in both disease-free and locoregional recurrence-free survival [76]. The second example is from a group at Memorial Sloan Kettering who piloted a novel approach of potential dose de-escalation in patients with human papillomavirus-related oropharyngeal cancer (30 ROC Trial) whereby tumor hypoxia was assessed by fluorine-18-labeled fluoromisonidazole positron emission tomography (FMISO-PET) pre-treatment and after 10-20 Gy + cisplatin with dose de-escalation to 30 Gy + cisplatin when there was either no hypoxia upfront on FMISO-PET or resolution of hypoxia on the mid-treatment FMISO-PET. In total, 15 of 19 patients were dose de-escalated with the remaining 4 patients treated to 70 Gy + cisplatin. Despite only 30 Gy of RT, 11 of 15 patients had pathologic CR (on neck dissection) and 2-year locoregional control was high at 94.4% [77]. The last example is from a group at the University of Michigan that used high b-value diffusion and dynamic contrast-enhanced (DCE) perfusion imaging midway through a course of therapy for poor prognosis HN cancer patients to define a high-risk subvolume that could be boosted with an additional 10 Gy. They performed a randomized phase II study with 41 control and 40 boosted patients and found a significant improvement in locoregional control in the high-dose group with no detectable increase in toxicity [78].

The field of RT has big challenges for implementing imaging biomarkers and using them for adaptive therapy but is on the cusp of doing so effectively. While it will still take some time to validate and incorporate these advanced imaging modalities into the clinical workflow, this should not diminish the enthusiasm for this process.

### 2.5. QA Issues with Widespread Implementation of Quantitative Imaging Modalities

The advancements in quantitative imaging have enabled the implementation of quantitative imaging in investigator-initiated and cooperative group clinical trials. Imaging has evolved from morphological imaging to functional imaging to molecular imaging to advanced data science approaches that use machine learning and deep learning methods to enable the detection of hidden imaging content. Seminal examples of quantitative imaging with a significant role to play in clinical trials include advanced imaging capabilities on PET systems using digital photon counting methods (NCT03387618), radiomics analysis [79], neural networks for dynamic imaging [80], and applying analysis methods directly to the acquired projection data rather than focusing on reconstructed images [81].

The incorporation of quantitative imaging in clinical trials poses unique QA challenges and requires attention to imaging-specific considerations when developing clinical trials. Several organizations have devoted significant resources to helping investigators manage quantitative imaging data in clinical trials. Example organizations that aid with quantitative imaging in clinical trials include the Quantitative Imaging Network (QIN) [82] as well as the Imaging and Radiation Oncology Core (IROC) within the National Clinical Trials Network (NCTN) [83]. The QIN published a web-based collection of software imaging tools along with detailed descriptions of each tool. IROC has put out best practices for imaging in NCTN trials that include performing real-time image quality reviews on a number of select cases, documenting commonly found image quality issues found during imaging review, working with IROC to prepare early protocol amendments that can address imaging QA challenges, and closely following up results to determine imaging compliance rates [83].

When considering incorporating quantitative imaging within clinical trials, several important factors need to be assessed that can guide investigators. The first aspect to consider is whether the trial is an observational trial or an interventional trial and whether the trial is imaging focused or therapeutic focused. Interventional trials that are imaging focused may have different requirements when compared to observational studies where the primary endpoint may not be imaging related. The investigators need to identify how imaging is being used in the trial and specifically identify whether imaging is being used for disease detection, disease stratification, response monitoring, disease progression, or adverse event reporting and design QA procedures relevant to the task that the quantitative imaging is being used for. An additional important aspect is to determine whether imaging is being used as an integral biomarker or an integrated biomarker; where an integral biomarker is defined as a biomarker that is essential to conducting the trial and must be performed in real time where an integrated biomarker is a biomarker used within the context of the trial to help answer questions pertinent to a particular drug, therapy, or intervention and may not require real-time evaluation. Based on the quantitative imaging needs of the trial, an important consideration is whether the trial-specific imaging is to be conducted locally or centrally. Local site-based tool use has the advantage of enabling real-time and real-world utilization and provides a clear pathway to broader clinical use. Central-based implementation (through IROC for example) requires only a single setup for a specific trial and has the advantage of providing a more controlled environment. For idealized quantification, imaging needs to be performed on identical systems and using the same imaging parameters (MRI sequences for example) whenever possible. Whether the imaging is conducted locally or centrally, trial-specific image credentialing is preferred to ensure consistent imaging parameters are used and a trial-specific imaging document is recommended. Investigators need to consider data sharing aspects of the quantitative imaging including what data is being shared, when is the imaging data being shared, and if any aspects of the data will be visible to the development team (which may be important in any image troubleshooting issues). An important aspect to consider is data quality expectations and specifically what Digital Imaging and Communications in Medicine (DICOM) labels are expected in the data. Finally, investigators should have an explicit plan describing how software bugs and troubleshooting will be managed.

## 3. Brief Conclusions

Quantitative imaging has evolved to take on an increased role in radiotherapy and clinical trials. A ‘Master Class’, was organized at the 2023 ASTRO meeting to highlight the latest advancements and challenges pertinent to using quantitative imaging in personalized radiation therapy. The current work covered seminal quantitative imaging methods that have been used to guide treatment including dual-energy CT and PET-CT for functional lung avoidance, functional MRI imaging for improved RT treatment of brain tumors, quantitative PET imaging for HN RT, and a summary of QA methods needed for functional imaging-based RT. It should be underlined that since the 2023 ASTRO Master Class on quantitative imaging, further advancements have been presented including clinical outcomes for functional avoidance [84], quantitative imaging in the surgical domain [85], and using PET scans to assess clinical outcomes [86]. Quantitative imaging has been incorporated into clinical care and has shown promising results that demonstrate improved disease cure rates and reduced toxicity for patients treated with radiotherapy. Current limitations of quantitative imaging in radiotherapy clinical care include that some methods remain investigational and lack widespread availability and that there is a relative lack of standardization for the functional imaging methods. Future work in quantitative imaging in radiotherapy will focus on improving the physiological accuracy of the functional imaging [87], improving the robustness of the methods such that any clinic (including clinics with limited prior experience) can adopt the methods [88], adoption of standardization practices, expanding the clinical data to include phase III, co-operative group clinical trials, and expanding applications to other disease sites (liver for example [89]) and other medical disciplines (surgical evaluation for example [90]). Quantitative imaging should be considered an important component in future multiscale modeling of patient care along with genomic and other clinical data.
